# Physical Conditioning Strategies for the Prevention of Concussion in Sport: a Scoping Review

**DOI:** 10.1186/s40798-021-00312-y

**Published:** 2021-05-17

**Authors:** Daniel Garnett, Jon Patricios, Saul Cobbing

**Affiliations:** 1 Physiotherapy Department, College of Health Sciences, University of KwaZulu Natal, Durban, South Africa; 2 Department of Physiotherapy, Faculty of Health Sciences, University of Pretoria, Pretoria, South Africa; 3 Wits Sport and Health (WiSH), School of Clinical Medicine, Faculty of Health Sciences, University of the Witwatersrand, Johannesburg, South Africa

## Abstract

**Background:**

Concussion in sports has received a great deal of media attention and may result in short and longer-lasting symptoms, especially in adolescents. Although significant strides have been made in the identification and management of concussion, less is known about the primary prevention of this condition. The aims of this scoping review are to (1) summarize the current research of physical conditioning strategies to reduce or prevent concussion incidence in individuals participating in sport, especially adolescents, and (2) to identify gaps in the knowledge base. Our research question was what is known from the existing literature about physical preparation strategies to reduce or prevent concussion in adult and adolescent sports?

**Methods:**

Three literature searches were conducted by information officers at two universities at six-month intervals, using five electronic databases (PubMed; WorldCat.org; Mendeley; EBSCOHost and Ovid MEDLINE). To increase the search range, subject experts were consulted and articles and reference lists were hand searched. A scoping review methodology identified eligible studies that analyzed physical preparation techniques on modifiable physical risk factors in athletes to reduce the incidence of concussion. The PRISMA-ScR checklist guided the reporting of the findings.

**Results:**

A total of 1414 possible articles were identified, after duplicates removed, and articles analyzed against the inclusion and exclusion criteria, only 9 articles qualified for analysis. Two articles were found from studying reference lists. Thus, a total of 11 articles were included in the final evaluation for the purposes of this study. Data are reported from mostly adolescent subjects participating in nine different sports from three countries. Findings are presented with specific reference to previously recognized modifiable risk factors of concussion which include neck strength, neck size, cervical stiffness, type of sport, and pre-activity exercises.

**Conclusions:**

There is limited research examining the physical preparation of athletes, especially in adolescents, to reduce or prevent concussion, and conflicting evidence in the few small sample studies that were identified. This scoping review identifies the research gap for a potentially vital modifiable risk factor, notably in the physical preparation of children and adolescents to reduce or prevent sports-related concussion.

**Supplementary Information:**

The online version contains supplementary material available at 10.1186/s40798-021-00312-y.

## Key Points

- Physical conditioning strategies may have beneficial effects on some modifiable risk factors for sustaining a sport-related concussion, although existing evidence is limited and conflicting.
- It is unclear which modes or dosage of physical conditioning strategies reduce the incidence or effects of sport-related concussion.
- Future studies to specifically assess the effects of both proprioceptive and dynamic strengthening of the neck musculature to reduce forces transmitted to the brain are recommended.

## Background

Sport-related concussion (SRC) has a high incidence affecting approximately four million people in the USA every year, mainly persons aged 7 to 19 years [1, 2], with potentially long-lasting adverse effects [3]. The incidence of concussion is highest in athletes participating in contact sports such as Rugby Union, American Football, basketball, wrestling, ice hockey, and soccer [4, 5]. In some sports, concussion is the most common injury, with the reported incidence increasing [6]. There is a heightened concern during important neurodevelopmental years as adolescents may demonstrate larger post-concussion neuropsychological deficits and symptoms compared to adults [7]. The negative symptoms of concussion may persist for a few days to several weeks, although in a small number of cases, symptoms may persist for longer than 3 months [8]. Public awareness of the potential adverse effects of repeated concussive exposures on the brain is increasing [3] and news reporting agencies in many countries have recently emphasized the need for improved interventions to reduce concussion incidence and improve player welfare [9–13]. International collaborative efforts have improved the understanding of SRC and the appropriate identification and management of children who have sustained an injury to the brain [14], but strategies for the primary prevention of concussion need further exploration to help ensure the health and safety of populations most at risk.

Trauma to the brain can result from direct and indirect contact events. Player-to-player contact (especially the tackle) is the most common scenario that results in concussion in many sports [4, 5, 15, 16], although contact with the ground [15] and equipment is also common [17, 18]. Due to the nature of contact and collision sports, physical contest between players is inevitable. Thus, research into making collision events safer is vital. Rule changes may have the greatest potential population health effect in lowering concussion in youth sport [19]. In soccer, head injuries were reduced following a single rule change for intentional elbow-head contact [20]. In Rugby Union (“rugby”), some law changes and educational initiatives have shown positive impacts in reducing the incidence of concussion and improving awareness [21]. Other law changes did not seem to reduce the incidence of concussion and increased the risk of sustaining a SRC to the tackler [22]; this research raised ethical concerns regarding participants’ informed consent and their right to withdraw. As a result, recommendations for substantiated research evidence and dialog should precede changes to the professional game [23]. Similarly in youth ice hockey, law changes have identified mixed results for injury risk [24, 25]. Current research which examines primary concussion prevention strategies in sport is, however, very limited [26].

The ability to minimize or modify identified risk factors may reduce the effect or incidence of concussions in children. Previously identified risk factors for concussion include: a history of concussions, level of education, age, level of competition, behavior, sex, unanticipated contact, neck stiffness, predisposing psychological factors, neck strength, and neck girth [4, 7, 15, 16, 18, 27–32]. Children are at greater risk for sustaining a concussion, for having increased severity of symptoms and for experiencing longer recovery times compared to adults, which may be attributable to muscular (especially neck) characteristics [7]. The ability to withstand forces indirectly or directly applied to the head has been proposed as a possible mechanism to reduce traumatic brain injury and concussion [33–37]. As a result, adults may be more resilient to the effects of head trauma as they have greater neck strength and neck girth to control the inertia of the head during potentially traumatic events.

Physical preparation exercises have proven successful in reducing the effects of less common and less serious injuries [38–42] and recent evidence highlights exercise as a treatment of SRC [43, 44]. However, little is known about how physical preparation exercises improve the primary prevention of concussion in populations at most risk. The primary aim of this scoping review is to achieve an in-depth and broad understanding of the current literature on physical preparation strategies specifically designed to reduce or prevent SRC, especially in adolescents. Secondary aims included identifying gaps in the existing evidence base and recommending areas for future research with a focus on physical conditioning to reduce the incidence of sport-related concussion.

## Methods

The methodological framework to identify literature for the current scoping review was developed by Arksey and O’Malley [45, 46]. This framework includes the following steps:

1. Identifying the research question
2. Identifying relevant studies
3. Study selection
4. Charting the data
5. Collating, summarizing and reporting the results
6. Optional consultation.

Finally, for reporting guidance of the results, the PRISMA-ScR checklist was used [47].

### Identifying the Research Question

The research question for this scoping review was *what is known from the existing literature about physical preparation strategies to reduce or prevent concussion in adult and adolescent sports?* The wide approach to this review was maintained to generate a breadth of coverage.

### Identifying Relevant Studies

**Fig. 1:**
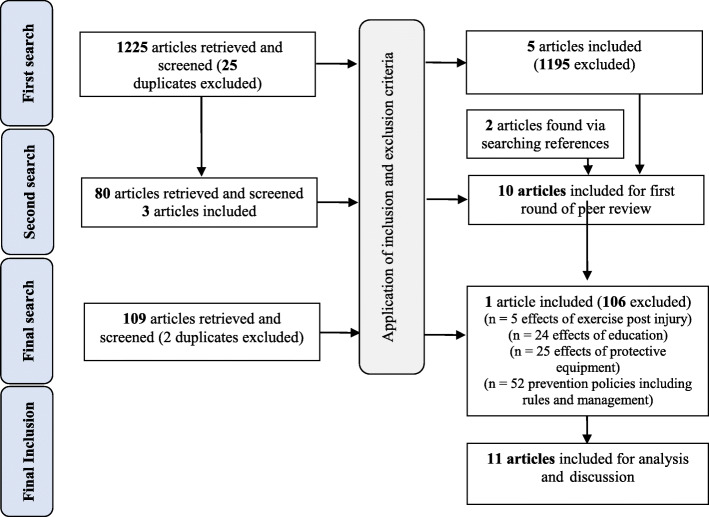
PRISMA-based article selection process

To comprehensively identify applicable search results of this scoping review, a three-round strategy involved searching for research evidence via different sources, including electronic databases, reference lists, and hand-searching journals (Fig. 1).

#### Electronic Databases, the Internet and Research Registers

The first electronic search was conducted on 9 September 2019 using keywords selected by the author team assisted by an Information Officer from University of KwaZulu Natal (UKZN) who guided the search strategy. The search strategy of UKZN’s electronic databases (PubMed; WorldCat.org and Mendeley) used the keywords and combinations of keywords as described in Additional file 1. Titles and abstracts were screened for inclusion after each consecutive search (Table 1), similarly, articles were excluded. Before submission on 30 April 2020, a second electronic search was conducted, using the same methods. Full articles were reviewed for a final decision on inclusion in this review. Following the peer-review process, a further electronic search was performed, to include the most recent research, on 1 December 2020 by an Information Officer from the University of Pretoria. This search was performed using the WorldCat.org, EBSCOHost and Ovid MEDLINE Databases using keywords as described in Additional file 2. The results were ranked by relevance and screened by title and abstract and retrieved 109 possible articles. This search was limited to English-only articles with publication dates between 1 January 2005 and 1 December 2020. The articles included in this review have been sourced by two information officers using five databases and reflect the current literature. The EndNote X9 online library was used for managing records and keeping track of articles. The EndNote Word Plugin is compatible with MS Word that the author used for the transcription of this manuscript.

**Table 1:** Article inclusion criteria

Criterion	Description
Publication type	Peer-reviewed Empirical research Methodology and results described in detail English language.
Publication date	Published between 1 January 2005 and 1 December 2020.
Participant characteristics	Adolescents and adult athletes or respective non-human models.
	Any participation in any sport or athletic activity during high school, college, professional, or amateur levels.
Injury characteristics	Sport-related mechanism of injury and/or, Modification of concussion risk factors and/or Diagnosed with a sport-related concussion (SRC) or mild traumatic brain injury using clinical diagnostic criteria: “Sport-related concussion is a traumatic brain injury induced by biomechanical forces. Several common features that may be utilized in clinically defining the nature of a concussive head injury include: • SRC may be caused either by a direct blow to the head, face, neck, or elsewhere on the body with an impulsive force transmitted to the head. • SRC typically results in the rapid onset of short-lived impairment of neurological function that resolves spontaneously. However, in some cases, signs and symptoms evolve over a number of minutes to hours. • SRC may result in neuropathological changes, but the acute clinical signs and symptoms largely reflect a functional disturbance rather than a structural injury and, as such, no abnormality is seen on standard structural neuroimaging studies. • SRC results in a range of clinical signs and symptoms that may or may not involve loss of consciousness*.”* [9]
Research design	Pre-injury intervention compared to the control group to reduce the incidence of concussion or risk factors for concussion. Post-injury performance on outcome measure(s) compared to the control group without acute concussion and injured athletes’ pre-injury baseline performance. Intervention to address modifiable physical risk factors for concussion.
Statistical information	Statistically significant findings regarding the reduction in risk factors or incidence of concussion, brain, and head injuries.

#### Reference List and Hand-Searching of Journals

To encompass a broader search, bibliographies, and reference lists of studies found in the electronic database were checked to discover further studies that match the inclusion criteria to be incorporated in this scoping review. Journals were hand-searched to identify articles that may have been overlooked in the electronic database and reference list searches.

### Study Selection

The information officers provided comprehensive lists of possible articles based on relevance and ranking. The lead author applied the inclusion and exclusion criteria to all these citations. All authors had an input on the analysis of the findings. Reflecting on the global advancement in the understanding of concussion in recent years, the preliminary search included articles less than ten years old. Due to the scarcity of results, this timeline was extended to 15 years. Thus, only those studies published between 1 January 2005 and 1 December 2020 were included. The search was limited to English language articles only, due to nearly all high impact and highly cited SRC articles being published in English and the cost and time involved in translating foreign material. However, there is a strong possibility that resources in other languages could have added to the information in this scoping review. Copies of the full articles were obtained. All articles that matched the exclusion criteria or did not match the inclusion criteria were removed (Table 1).

#### Study Exclusion Criteria

Articles were omitted if they were older than 15 years, not peer-reviewed, not in the English language, and were considered expert opinion only.

### Charting the Data

The data were interpreted by sorting material according to key issues and themes using Microsoft Excel. Together, these data formed the basis of the analysis. The information was recorded as follows:

- Author(s), year of data collection, study location, sport
- Intervention type, and comparator (if any); duration of the intervention
- Study populations (carer group; care recipient group)
- Aims of the study
- Methodology
- Outcome measures
- Important results

## Results

### Framework Stage 5: Collating, Summarizing, and Reporting the Results

The results of this search were summarized and combined in the data extraction table (Table 2). The authors interpreted and analyzed the results within the broader context of participant health in sports activities.

**Table 2:** Articles included in this scoping review

Study	Year*	Duration and location	Sport	Intervention	Population	Aims	Methods	Outcome	Results
Attwood et al. [48]	2015/2016	Seven 6-week phases (294 days) in the UK	Rugby Union	Proprioceptive, strengthening and mobility exercises with progressive structure targeting the lower limb, shoulder, head, and neck.	81 community rugby clubs (25.5 ± 5.6 years, 1.86 ± 0.073 m, 94.4 ± 13.9 kg).	To investigate the efficacy of a rugby-specific movement control program to reduce injury risk in adult men’s community rugby union players.	First team match exposure, exercise program compliance and match injuries were reported on a weekly basis using standardized forms. Injury burden (number of days absence per 1000 player match-hours) and 90% CIs were estimated vis-à-vis for primary and secondary outcome measures. Injury incidence was estimated as the number of injuries per 1000 player match-hours.	A likely beneficial difference in targeted injury incidence (rate ratio (RR), 90% CI = 0.6, 0.4 to 1.0) with a 40% reduction in lower limb incidence (RR, 90% CI = 0.6, 0.4 to 1.0) and a 60% reduction in concussion incidence (RR, 90% CI = 0.4, 0.2 to 0.7). Clubs with highest compliance demonstrated very likely beneficial 60% reductions in targeted injury incidence (RR, 90% CI = 0.4, 0.2 to 0.8) and targeted injury burden (RR, 90% CI = 0.4, 0.2 to 0.7).	No clear effects on overall injury outcomes however, the intervention group had a significant reduction in the incidence of lower limb injury and concussion. Reductions were greater in clubs who performed the exercises more than those that did not.
Becker et al. [49]	Year of data collection not stated	Six weeks duration including pre- and post-intervention testing phases in Germany	Male soccer	Two training intervention groups and one control group. The training intervention included three resistance neck exercises for 6 weeks (2×/week). Rubber band strength was increased after every 4 sessions.	Thirty-three active male soccer players (20.3 ± 3.6 years, 1.81 ± 0.07 m, 75.5 ± 8.3 kg).	To analyze the effect of a 6-week strength training for the neck flexors and extensors on the acceleration of the head during standing, jumping, and running headers in soccer on a stationary pendulum header	Isometric maximum voluntary contraction (IMVC) measured by a telemetric Noraxon DTS force sensor accelerometer (Noraxon, Scottdale, USA; size: 22 × 16 × 7 mm; weight: 2.8 g; frequency: 1500 Hz, filter: lowpass 500 Hz) fixed in the occipital area of the head. Participants were exposed to two rounds of testing, each round consisting of pre-fatigue test and a post fatigue test. Fatigue was achieved by the Bourban test. The two pre-fatigue tests (standing, jumping, and running header) and the two post-fatigue tests (post-jumping and post-running header) were compared	There was no significant change of the IMVC over time between the groups (*F* = 2.265, *p* = .121). Head acceleration was not reduced significantly for standing (IG1 0.4 ± 2.0, IG2 0.1 ± 1.4, CG − 0.4 ± 1.2; *F* = 0.796, *p* = 0.460), jumping (IG1 − 0.7 ± 1.4, IG2 − 0.2 ± 0.9, CG 0.1 ± 1.2; *F* = 1.272, *p* = 0.295) and running (IG1 − 1.0 ± 1.9, IG2 − 0.2 ± 1.4, CG − 0.1 ± 1.6; *F* = 1.050, *p* = 0.362) headers as well as after fatigue of the trunk musculature for post-jumping and post-running headers over time between IG1, IG2, and CG	The presumed preventive benefit of this 6-week strength training of the neck flexors and neck extensors could not be confirmed statistically. The authors recommend a training period of at least 8 weeks.
Caccese et al. [50]	Year of data collection not stated	A single round of testing included 1000 simulations of standing headers in experimental test conditions in North America	Boy’s and girls’ soccer	Participants performed a series of 12 standing headers to a target located approx. 2 m in front of them using soccer balls projected (initial velocity = 11.2 m/s at 40° over approximately 12 m) using a machine (JUGS, Tualatin, OR, USA)	One hundred soccer players: 42 males, 58 females, 17.1 ± 3.5 years, 168.5 ± 20.3 cm, 61.5 ± 13.7 kg, and 13.3 ± 3.0 years of soccer participation	To determine the relationships between head and neck size, neck strength and heading technique on head acceleration magnitudes in youth, high school, and collegiate male and female athletes during purposeful soccer heading	Participant height, weight, and head and neck anthropometrics were measured. Isometric strength measurements of the sternocleidomastoid and upper trapezius muscles using a hand-held dynamometer. EMG activity of Sternocleidomastoid and upper trapezius measured using the Trigno™ Wireless System (Delsys Inc., Natick, MA, USA). Heading kinematics were determined using an 8-camera motion capture system (Motion Analysis Corporation, Santa Rosa, CA, USA). Head accelerations were also recorded.	Head mass significantly predicted peak rotational acceleration (β = − 0.404, *p* = 0.034). The sternocleidomastoid strength significantly predicted peak linear and rotational acceleration (linear β = − 1.544, *p* = 0.012; rotational β = − 0.117, *p* = 0.018). Technique-related predictors did not significantly predict peak linear acceleration (*R*^2^ = 0.066, *F* (6,87) = 1.029, *p* = 0.412, ƒ2 = 0.07) or peak rotational acceleration (*R*^2^ = 0.047, *F* (6,87) = 0.730, *p* = 0.627, ƒ2 = 0.05)	Greater head and neck size predicted lower peak linear and rotational accelerations. A soccer player with smaller head mass, neck girth, and neck strength may sustain greater head acceleration. The authors recommend that anthropometric and neck strength measures should be considered when determining readiness to begin soccer heading
Collins et al. [27]	2010–2011	One academic year in North America	Boy’s and girls’ soccer, basketball, and lacrosse	Baseline testing of strength and physical measurements taken at preseason and correlated with reported concussion incidence and athletic exposure data	Fifty-one high schools in twenty-five states participated. 6704 high school athletes in boys’ and girls’ soccer, basketball, and lacrosse	To develop and validate a cost-effective tool to measure neck strength. To determine if this tool is applicable by athletic trainers, and to determine if anthropometric measurements can predict concussion risk	A hand-held dynamometer, a hand-held tension scale, Velcro closure head band with D-rings, and a cloth measurement tape to measure head and neck circumference, neck length, and four measurements of neck strength for all athletes participating in school-sports	Neck strength, sex, and sport were significant predictors of concussions. After adjusting for sex and sport, overall neck strength remained a significant predictor of concussion. For every one-pound increase in neck strength, odds of concussion decreased by 5%	Smaller mean neck circumference, smaller mean neck to head circumference ratio, and weaker mean overall neck strength were significantly associated with concussion
Eckersley et al. [51]	Year of data collection not stated	A single round of testing included 192 simulations spanning the experimental test conditions in North America	Non-human simulations of impacts to the head during possible participation in baseball and American Football	Kinematic data of neck models were recorded for impacts to 8 different locations on the head in four different scenarios in six different neck conditions	Impacts using models from Duke University (DUHNM) and the National Crash Analysis Center (NCAC) at George Washington University	To investigate the role of cervical muscle strength in blunt impact head kinematics and the biofidelity of common experimental neck conditions	Four impact scenarios were created. The first scenario simulated a baseball impacting a bare head. The second and third scenarios simulated helmet to helmet collisions with shorter and longer durations respectively. The fourth scenario modeled a lesser helmet to helmet impact force	Kinematic differences from impact location and strength can be ten times greater than cervical muscle activation forces. Relaxed neck conditions showed lowest peak resultant angular acceleration values for 65% of impacts. Extensor neck conditions showed highest peak resultant angular acceleration values	Results suggest that increased cervical muscle force does not influence short term (< 50 ms) head kinematics. Impact location and magnitude influence head kinematics more than cervical muscle state
Eckner et al. [52]	Year of data collection not stated	A single round of testing with three trials in each head position under both muscle activation conditions in North America	Soccer, ice hockey, American Football, martial artists, wrestling and lacrosse	Maximum isometric neck strength was measured using a loading apparatus which applied impulsive test forces to athletes' heads during baseline and anticipatory cervical muscle activation conditions	Forty-six athletes (24 males; 22 females); age range 8–30 years.14 males and 12 females in high school or younger,10 males and females each from college or older. All from a broad range of competitive levels	To determine the influence of neck strength and muscle activation status on resultant head kinematics following impulsive loading	Wrestling headgear was attached to an adjustable cable with an in-line force transducer cable. Head kinematics were measured using an Optotrak motion capture system for peak force values in head flexion, extension, right lateral flexion, or left axial rotation. Demographic and anthropomorphic measurements were taken. Sonographic cross-sectional area of the right sternocleidomastod muscle was collected	Significant effects for neck strength and cervical muscle activation status across all directions of motion. All neck strength and cervical muscle activation effects remained significant when adjusting for age and sex. Neck circumference and sternocleidomastoid cross-sectional area both had significant effects across all directions of motion, which remained significant when adjusting for age and sex	Greater neck strength attenuates the head's dynamic response to external forces in all planes of head motion and across the age spectrum in athletes of both sexes
Hislop et al. [53]	2015	One playing season from August to December 2015 in the UK	Rugby Union	Balance training, whole-body resistance training, plyometric training, and controlled rehearsal of landing and cutting maneuvers	Three thousand one hundred eighty-eight rugby players aged 14–18 years. Twenty schools in each of the intervention and control groups	To determine the efficacy of an exercise program in reducing injuries in youth rugby players and to investigate the effect of program dose on injury measures	Pre-activity exercises for each the intervention and control groups had four phases with increasing difficulty. Coaches recorded training exposure, match exposure, program compliance, and the return-to-play date. School medical staff recorded the injury location and diagnosis	Overall match injury incidence (injuries/1000 player-hours) and burden (days lost/1000 player-hours) rates acted as dependent variables, with further stratification by injury location and event.	Intention-to-treat analyses revealed that the intervention program substantially reduced upper limb injury burden and concussion incidence compared with the control program.
Lisman et al. [54]	Year of data collection not stated	8 weeks duration in North America	American Football	Isometric cervical resistance-training program of three sets of 10 repetitions of neck extension, flexion, and right and left lateral flexion at 60–80% of 10 repetition maximum (RM), 2–3×/week	Sixteen male participants (age 21.6 ± 2.8 years)	To examine the effects of an 8-week isometric cervical resistance program on the electromyographic (EMG) activity of the sternocleidomastoid (SCM) and upper trapezius (UT) as well as the kinematics of the head and neck in response to a American Football tackle	Isometric cervical strength, neck girth, and both the EMG and kinematic responses of the head and neck during tackling were measured before and after training. Kinematic data were gathered using a ViconNexus® 3D motion capturing system.	Strength measurements in extension and left lateral flexion were statistically significant (73.64–78.81 kg, *p* = 0.004: 25.49–27.92 kg, *p* = 0.033). No significant difference was noted for neck girth. No significant effects for peak linear or angular head acceleration, head-cervical segment angular displacement, or time to peak angular acceleration. No influence on the EMG or kinematic responses	This 8-week cervical resistance training program had no effect on the EMG activity of the neck musculature and kinematics of the head and neck in response to a American Football tackle
Mansell et al. [55]	2005	Eight weeks duration in North America	Soccer	Pre-test and post-test study with a control group and intervention group who performed resistance exercises	Thirty-six Division I collegiate soccer players (17 men, 19 women)	To determine the effect of an 8-week resistance training program on head-neck segment dynamic stabilization	8-week cervical resistance training program of 3 sets of 10 repetitions at 55% to 70% of a 10-repetition maximum 2×/week. Participants in the control group performed no cervical resistance exercises	Head-neck segment kinematics and stiffness. Electromyographic activity of the upper trapezius and sternocleidomastoid muscles during force application to the head, and isometric neck strength.	Increases in isometric strength and girth were found in the intervention group. Training did not enhance the head-neck segment dynamic stabilization.
Mihalik et al. [33]	2011	One playing season duration in North America	Ice hockey	Instrumented helmets collected head impact biomechanics.	Thirty-seven volunteer ice hockey players	To determine the effect of cervical muscle strength on head impact biomechanics	Preseason cervical muscle strength was measured using isometric “break tests” with a hand-held dynamometer	Dependent variables included linear and rotational head accelerations.	Players with greater static neck strength did not experience lower resultant head accelerations
Schmidt et al. [30]	2014	One off testing procedure in preseason and players followed for a season in North America	American Football	Baseline testing with surveillance of head impact biomechanics	Forty-nine high school and collegiate American Football players (34 high school, 15 collegiate), free of prior head/neck injuries or pain	To determine whether American Football players with stronger, larger, and stiffer cervical muscle characteristics at preseason had reduced odds of sustaining higher magnitude head impacts	Preseason testing: isometric strength using the HUMAC NORM system. EMG during cervical perturbation was captured. Ultrasonic cross-sectional area was obtained from images using a 7-MHz linear array transducer. Head impact biomechanics captured using the Head Impact Telemetry System	Players had equal odds for moderate and severe head impacts regardless of cervical muscle strength. Players with larger Sternocleidomastoid, Semispinalis capitis, and composite muscle areas had increased odds, players with stiffer necks during anticipated forced extension and composite stiffness had reduced odds.	The findings did not show that players with stronger and larger neck muscles mitigate head impact severity. Greater cervical stiffness and less angular displacement after perturbation reduced the odds of sustaining higher magnitude head impacts.

"Year*" indicates the year of data collection for the respective study

## Characteristics of Included Samples

The 11 articles included in this review comprised of studies conducted only in the Northern Hemisphere, 8 in North America, 1 in Germany, and 2 in the UK [27, 30, 33, 48–55]. One study used non-human neck model simulations [51]. The human participants of the remaining included studies varied significantly in age. One study included only adult males (men aged 25.5 ± 5.6) [48]. Three studies included collegiate participants, (men aged 19.21 ± 0.918; women aged 19.16 ± 0.898 years) [55] (males aged 20.3 ± 3.6) [49] (men aged 21.6 ± 2.8) [54], three studies included high school and collegiate participants (high school 16.6 ± 0.9, collegiate 20.5 ± 1.4 years) [30]; (16.3 ± 5.0 years for males and 15.0 ± 4.4 years for females) [52] (males and females 17.1 ± 3.5) [50], and three studies included only high school participants (intervention 16.0 ± 1.2 years; control 15.9 ± 1.1) [53]; (15.0 ± 1.0 years) [33]; (girls and boys with no age descriptions) [27].

With regard to sports codes discussed in the selected articles [27, 30, 33, 48–55], two studies reported exclusively on Rugby Union (men; sex unspecified) [48, 53], another exclusively ice hockey (sex unspecified) [33], two studies on American Football (sex unspecified) [30] (males only) [54], two studies focused on men’s and women’s soccer [50, 55] and one study on men’s soccer only [49]. Three studies reported on multiple sports codes, namely, boys’ and girls’ soccer, basketball, and lacrosse [27], soccer, ice hockey, American Football, wrestling, lacrosse, and martial arts [52] and baseball and American Football [51].

Mansell et al. (2005) [55] reported on the effect of an 8-week isotonic resistance training program on head-neck segment dynamic stabilization in a small sample of men’s and women’s soccer players [55]. The training program consisted of 3 sets of 10 repetitions of neck flexion and extension at 55% to 70% of their 10-repetition maximum, two times a week. Measurements included anthropometric assessments, head-neck segment kinematics and stiffness, electromyographic activity of the upper trapezius and sternocleidomastoid muscles during force application to the head, and neck flexor and extensor isometric strength. Although the intervention group showed a 15% improvement in isometric neck flexor strength, no kinematic, electromyographic, or stiffness training effects were seen. In female intervention group participants, isometric neck extensor strength increased by 22.5%, and neck girth increased by 3.4%. Female soccer players demonstrated less head-neck segment length (7%) and less head-neck segment mass (26%) than men. The researchers concluded that regardless of the improvements in neck isometric strength and increases in neck girth, the resistance training protocol used in this study did not increase head-neck segment dynamic stabilization during force application in soccer players [55].

Schmidt et al. (2014) reported on the incidence and nature of head impact biomechanics using the Head Impact Telemetry System in a small sample of American Football players who completed preseason cervical testing of isometric neck strength, electromyography, muscle size, and response to cervical perturbation [30]. The reported findings showed the likelihood of sustaining higher magnitude head impacts was reduced in players with greater cervical stiffness and who experienced a smaller amount of angular displacement after impact. The results of this study showed that players with stronger lateral flexors and composite cervical strength had increased likelihood (1.75×) of receiving moderate head impacts rather than mild impacts, compared with weaker players. Similarly, players who developed faster torque in cervical extension had twice the likelihood of receiving severe head impacts (odds ratio [OR], 2.10; 95% CI, 1.08–4.05) rather than mild head impacts. However, players with greater cervical stiffness had reduced likelihood of sustaining both moderate (OR, 0.77; 95% CI, 0.61–0.96) and severe (OR, 0.64; 95% CI, 0.46–0.89) head impacts compared with players who demonstrated less cervical stiffness. The authors conclude that the study’s findings showed that greater cervical stiffness reduced the likelihood of sustaining higher degree head impacts. Further, the results of this study do not support that stronger and larger neck muscles reduce the severity of head impacts [30].

Lisman et al. (2012) examined the effects of an 8-week isoinertial cervical resistance training program and the electromyographic (EMG) activity of neck muscles and the kinematics of the head and neck in response to a American Football tackle [54]. The results of the study showed modest increases in isometric strength in cervical extension (7%) and left lateral flexion (10%), but the program had no influence on the EMG responses of neck muscles, peak linear, or angular head accelerations during tackling. This authors conclude that this 8-week isoinertial cervical resistance training program did not increase dynamic stabilization of the head and neck during a American Football tackle [54].

Mihalik et al. (2011) evaluated the effect of cervical muscle strength on the head after an impact by collecting data from accelerometer instrumented ice hockey helmets throughout a playing season [33]. A small sample of players’ cervical isometric strength measurements were recorded using a hand-held dynamometer (Model: 01163; Lafayette Instrument, Co, Lafayette, IN) in the preseason. Muscle strength testing involved two practice trials performed before three test trials (of 3 s duration) for each direction of motion, with a 30 s rest period between trials. The maximum forces for each of the three test trials were averaged and normalized to the player’s body mass. These data were compared with head biomechanics from the collected helmet data (Head Impact Telemetry System). The authors identified significant differences in cervical muscle strength between the participants. However, no differences were recorded in peak linear (*P*_Lin_) or peak rotational acceleration (*P*_Rot_) for the anterior neck flexors (*P*_Lin_ = 0.399; *P*_Rot_ = 0.060), anterolateral neck flexors (*P*_Lin_ = 0.987; *P*_Rot_ = 0.579), cervical rotators (*P*_Lin_ = 0.136; *P*_Rot_ = 0.238), posterolateral neck extensors (*P*_Lin_ = 0.883; *P*_Rot_ = 0.101), or upper trapezius (*P*_Lin_ = 0.892; *P*_Rot_ = 0.689). The authors concluded that the findings of this study do not support that cervical muscle strength is a factor in modifying head impact severity [33].

Collins et al. (2014) reported on a large sample of adolescent athletes (*n* = 6662) over a full academic year in multiple contact sports, namely soccer, basketball, and lacrosse [27]. In this study, the researchers developed and validated a cost-effective tool to measure neck strength in these athletes and found a high correlation (0.83 to 0.94 for the four neck strength measurements—all *p* values < 0.05) between the hand-held dynamometer and tension scale measurements. High inter-tester reliability was observed between different athletic trainers (ATs). In the second part of the study, AT’s recorded athletic anthropometric measurements, exposure, and injury data on the internet-based data collection tool developed for the National High School Sports-Related Injury Surveillance Study. Athletes were prospectively monitored for sustaining a concussion from 2010 to 2011. The results showed that the rate of concussion in the three contact sports was higher in adolescent girls when compared to adolescent boys (4.9 per 10,000 athlete exposures in girls and 2.5 per 10,000 athlete exposures in boys), with soccer having the highest rate of concussion (5.2 per 10,000 athlete exposures) followed by lacrosse (3.7 per 10,000 athlete exposures) and basketball (2.3 per 10,000 athlete exposures). Girls had an increased likelihood of concussion overall (OR = 1.8, 95 % CI 1.36–2.49) and in basketball (OR = 2.7, 95% CI 1.53–4.71) and soccer (OR = 1.8, 95% CI 1.17–2.69). However, no difference was identified between girl and boy lacrosse players (OR = 1.0, 95% CI 0.44–2.10). The researchers reported that a smaller mean neck circumference, smaller mean neck to head circumference ratio, and weaker mean overall neck strength were significantly associated with concussion. Overall, sex (*p* < 0.001), sport (*p* = 0.007), and neck strength (*p* < 0.001) were found to be significant predictors for sustaining a concussion. Specifically for neck strength, the authors reported that for every 1 lb increase in neck strength, the likelihood of concussion decreased by 5% (OR = 0.95, 95% CI 0.92–0.98) [27].

Hislop et al. (2017) evaluated the effects of a prescribed series of progressive warm-up exercises in a cluster-randomized trial of adolescent rugby players injuries over one season [53]. The specific particulars of the intervention and control group exercises are not described in detail and the reader is directed to a previous study by the author for more information [56]. The intervention group exercises consisted of isometric neck exercises, whole-body resistance training, plyometric training, and landing and cutting running movements. These exercises were to be completed in the initial fifteen minutes of training and before every match, although the authors report that certain exercises were “withdrawn when the program is performed prior to matches” [56]. The control group exercises were structurally indistinct to the intervention program and consisted of exercises that were considered “best practice” in schools’ rugby including a running-based warm-up, dynamic stretching, wrestling, mobility, speed, and agility-related exercises [56]. School coaches recorded training exposure, player injury details, match exposure, and program compliance on paper-based or electronic report forms. School medical staff recorded the injury location and diagnosis. The intention-to-treat analyses indicated unclear effects of the trial arm for overall match injury (incidence rate ratio [RR] = 0.85, burden RR = 0.83) and match contact injury (incidence RR = 0.85; burden RR = 0.88). The researchers conclude that the players in the intervention group reported substantially reduced incidence of upper limb injury and concussion. Further, teams that completed the intervention program three times per week reported substantial reductions (72%) in overall match injury incidence (RR = 0.28, 0.14 to 0.51) and concussion incidence (RR = 0.41, 0.17 to 0.99) compared with the control program [53].

Similarly, Attwood et al. (2018) investigated the effects of a movement control program to reduce injury risk in rugby union players [48]. The intervention program involved proprioceptive, mobility, and strengthening exercises targeted at the lower limb, shoulder, head and neck over seven 6-week progressive phases. The control program involved dynamic stretching and non-targeted resistance exercises in a similar progressive structure to the intervention program. Participants were blinded to which program they received. Each participating club nominated an individual who was trained to deliver the program to the players, and a representative to record first team match exposure, exercise program compliance, and match injuries on a weekly basis, using standardized forms. No clear effect was identified for the intervention program using intention-to-treat analysis for overall injury burden, overall injury incidence or severe injury incidence. However, concussion incidence (1.2 vs 3.4 injuries/1000 player match-hours) and concussion burden (38 vs 102 days/1000 player match-hours) was 60% lower in the intervention group compared with the control group. Lower-limb injury incidence was also 40% lower for the intervention group over control group (3.3 vs 5.2 injuries/1000 player match-hours) although shoulder injury incidence (1.7 vs 1.0 injuries/1000 player match-hours) and injury burden (68 vs 45 days/1000 player match-hours) were higher for the intervention group. Further, clubs in the intervention group that had a greater compliance (≥ 85% to < 85% of possible sessions) indicated a likely beneficial 50% reduction in targeted injury burden [48].

Eckner et al. (2014) assessed the influence of neck size, neck strength, rate of force development, and muscle activation on head kinematics following loading in multiple planes [52]. The participants in this cohort comprised of a broad range of ages, competitive levels, and sporting codes. The results of this study showed greater isometric neck strength and anticipatory activation to be independently associated with decreased head peak linear velocity and peak angular velocity after impulsive loading across all planes of motion (all *p* < .001). Further, neck circumference and sternocleidomastoid cross-sectional area were also significant (*p* < .001) in all planes of motion and remained significant when adjusted for age and sex (*p* < .001). This study reports that superior neck strength and anticipatory muscle activation are individually associated with a decreased kinematic response to impulsive forces applied to a subject's head [52].

Caccese et al. (2018) aimed to identify factors that contribute to head acceleration during soccer heading. This study utilized anthropometric measurements, isometric strength and electromyography of muscles of the neck and upper torso and kinematics of the head during active soccer heading in seasoned soccer players [50]. The authors reported that the results suggest that greater head and neck size predicted lower peak linear and rotational accelerations. The results further showed that neck strength, specifically of the sternocleidomastoid muscle predict peak linear (β = − 1.544, *p* = 0.012) and peak rotational (β = − 0.117, *p* = 0.018) accelerations of the head. Technique-related predictors did not predict the same during soccer heading [50].

Eckersley et al. (2019) reported on the effects of cervical muscle strength on head kinematics using validated neck model simulations [51]. This study examines plausible impacts to the head for different athletic scenarios, namely impact from a ball to the bare head in major league baseball and impacts between opposing player American Football helmets. The authors report that no consistent effect to the injury metrics for sport-related concussion (SRC) can be seen by changing neck muscle force in models. The results did show that tensed muscle activation conditions resulted in higher peak resultant angular acceleration values compared to relaxed muscle activation conditions. The authors conclude that impact location and impact scenario were greater determinants of SRC injury metrics than the protective capacity of cervical muscle activation. The results of this study do not support the hypothesis that greater cervical muscle force influences head kinematics during impact scenarios and neck strengthening programs and exercises will do little to reduce the risk of concussion [51].

In the most recently published study included in this scoping review, Becker et al. (2019) explored the effects of a 6-week strength training program on head acceleration during three different variations of headers on male soccer players [49]. An interesting inclusion in this study design is that the researchers attempted to fatigue the trunk muscles of the participants to decouple the head-neck-torso alignment, thus resulting in an increased acceleration of the head. The results did not show a significant difference between the strength measurements of the control and intervention groups (*p* = 0.055). Neck flexion strength improved for all the groups, including the control group, who did not perform extra neck exercises. Neck extension strength improved for one of the intervention groups (youth team) but decreased in the other intervention (adult team) group, and in the control (mixed) group. The authors state that these results do not support the hypothesized preventative benefit of neck strengthening [49].

### Framework Optional Stage: Consultation Exercise

As an additional stage in scoping review methodology as recommended by Arksey and O’Malley [45], two International Concussion Societies were consulted (International Concussion Society https://www.concussion.org/contact/ and The Center for Disease Control and Prevention, U.S. Department of Health and Human Services) for further possible information. These organizations were contacted due to their location as most of the studies identified in this review were conducted in North America. The literature sourced from this optional exercise provided insight into the broader discussion of concussion although the provided literature did not satisfy the inclusion criteria for this scoping review.

## Discussion

### Summary of Evidence

This study aimed to summarize the current research on physical conditioning strategies to address specific modifiable risk factors in the prevention of sports-related concussion. Secondly, this study aimed to identify the gaps in the knowledge base regarding physical conditioning strategies to address these specific modifiable risk factors.

The studies included in this scoping review provide a lack of generalizability to the broader sports-playing populations for several reasons. Firstly, 6 of the 11 studies have small sample sizes (range *n* = 16–49). Secondly, 3 of the 10 studies do not describe the sex of the sampled participants. Thirdly, the study with the largest sample size does not specify the ages of its participants. Fourthly, there are significant disparities in age when comparing the results of participants (range 10.6–31.1 years) (Table 3), and previous research has identified significant differences in neck strength in adolescent athletes compared to adult athletes. Further, one study incorporated simulated models instead of human participants. Lastly, the studies included in this review only represent populations in North America, England and Germany and may not be representative of the global community.

**Table 3:** Identified study details and findings for modifiable risk factors

Study	Modifiable risk factors for concussion	Sport	Age range (years)	Sample number	Sex
Mansell et al. 2005 [55]	Neck strength—No	Soccer	18.26–20.13	*n* = 36	Male and female
Mihalik et al. 2011 [33]	Neck strength—No	Ice hockey	14.0–16.0	*n* = 37	Unspecified
Lisman et al. 2012 [54]	Neck strength—no	American football	18.8–24.4	*n* = 16	Male
Collins et al. 2014 [27]	Neck strength—yes Neck size—yes	Soccer Basketball Lacrosse	Unspecified	*n* = 6704 (total)	Male and female
Eckner et al. 2014 [52]	Neck strength—yes Neck size—yes	Soccer, ice hockey, American football, martial arts, wrestling, and lacrosse	10.6–21.3	*n* = 46	Male and female
Schmidt et al. 2014 [30]	Neck strength—no Neck size—no Cervical stiffness—yes	American football	15.7–21.9	*n* = 49	Unspecified
Hislop et al. 2017 [53]	Pre-activity movement exercise program—yes	Rugby Union	14.8–17.2	*n* = 3188	Unspecified
Caccese et al. 2018 [50]	Neck size—yes Neck strength—yes	Soccer	13.6–20.6	*n* = 100	Male and female
Attwood et al. 2018 [48]	Pre-activity movement exercise program—yes	Rugby Union	19.9–31.1	*n* = 41 clubs (participants unknown)	Male
Eckerlsey et al. 2019 51]	Neck strength—no	Simulated baseball, simulated American football	Not applicable	Not applicable	Not applicable
Becker et al. 2019 [49]	Neck strength—no	Soccer	16.7–23.9	*n* = 33	Male

### Neck Strength

This review identified conflicting evidence in a minimal number of studies regarding the effect of neck strength as a risk factor for concussion in adolescent athletes [27, 30, 33, 49–52, 54, 55] (Table 3). One large study showed that neck strength is a significant predictor of concussion [27]. Further, greater neck strength has been shown to attenuate head kinematics during unanticipated and anticipated loading of the head [52]. Other studies reported on isometric exercises, which have been shown to increase neck strength, especially in women, although this type of exercise does not show improvements in dynamic stabilisation of the head [55] or in modifying head impact severity [33]. This is supported by Eckersley et al. (2019), who state that neck strengthening exercises are not effective in reducing concussion risk and cervical muscle force does not influence head kinematics after impact [51]. Further, Lisman et al. (2012) and Becker et al., (2019) did not find preventative benefits of neck strengthening to reduce head acceleration forces [49, 54]. One study found that players with stronger cervical musculature were at higher risk of receiving more severe impacts to the head, possibly as a result of risk compensation which theorizes that players accept a certain level of risk and adapt their behavior based on perceived risk until their accepted level of risk is reached again [30] Contrastingly in the largest study in this review, isometric neck exercises were shown to significantly reduce the incidence of concussion in Rugby Union players [53]. Caution should be exercised with this finding as the authors do not measure neck strength or girth and only speculate that their exercises preserved neck function and potentially reduced forces to the brain [53].

Due to the dynamic nature of a concussive event, it is unlikely that strengthening a muscle at a fixed length without movement (isometrically) would achieve the desired response of reducing forces to the brain. The methodology of testing isometric strength in these studies is probably due to the difficulty of testing dynamic neck strength. Based on the studies reviewed here and the current understanding of SRC, future research should assess the effects of both proprioceptive and dynamic (especially eccentric, ballistic and plyometric) strengthening of the neck musculature to reduce forces transmitted to the brain. Ideally, isotonic strength testing through an athlete’s available neck range of motion in all available planes, including rotation in a manner to prepare the body to withstand shearing forces, would provide valuable information towards preparing athletes for participation in sport.

### Neck Size

Four studies addressed the effect of neck size as a modifiable risk factor for concussion and reported opposing views. The first study assessed a small sample of American Football players and concluded that players with larger cervical musculature might be at a greater risk of sustaining a concussion, possibly as a result of increased risky technique due to their perception of being more protected from injury [30]. The second study reported on multiple contact sports with a large sample of male and female participants and concluded that a smaller average neck circumference and smaller average neck to head circumference ratio were significantly associated with concussion [27]. The third study reported greater neck circumference and sternocleidomastoid muscle cross-sectional area, in particular, reduced peak linear and peak angular velocity of the head during impulsive loading [52]. The fourth study reported on male and female soccer players and the results show that greater neck girth significantly predicted lower peak linear and rotational head acceleration [50].

### Cervical Stiffness

Cervical stiffness in this context relates to the ability of the neck musculature and osteoligamentous structures to withstand displacement and has been proposed as a potential preventative strategy for reducing the risk of concussion, as well as the severity of sub-concussive trauma [30]. In this review, players with greater cervical stiffness had reduced likelihood of sustaining both moderate and severe head impacts compared with players who demonstrated less cervical stiffness.

### Type of Sport

The limited sporting codes included in the identified studies are considered “contact sports” and were most likely recognized for the increased risk of concussion in the participating athletes [57]. It should be noted that although there is a potential risk of concussion in contact sports, there is also a potential risk of concussion in non-contact sports codes and in non-contact events, such as cheerleading, volleyball, track and field, and softball [4].

### Whole-Body Pre-activity Exercises

Two impactful studies reported a substantial reduction in concussion incidence as a result of pre-participation whole body exercises [48, 53]. There are some limitations to these studies which have attracted criticism [58]. In both studies, with similar methods, the researchers did not test for baseline measurements (for example, cervical strength or cervical stiffness) and were only able to speculate as to the reasons for the reported changes. Both sets of authors suggest that the basic isometric neck exercises may have preserved neck function over the playing season and prevented concussion by dissipating forces applied to the brain [48, 53]. However, Attwood et al. cite a study with numerous limitations to support this statement [48, 59, 60]. Further, Hislop describes a progression through the advancing phases which does not allow sufficient time, frequency, or load required to improve strength [61, 62]. The scope of these studies do not allow adequate identification of the specific exercises which may be responsible for the substantial reduction in concussion incidence; however, the findings reported by Hislop et al. and Attwood et al. are substantial using relevant and large sample sizes [48, 53, 56]. These findings encourage future research as they are in stark contrast to previous studies of similar exercises with more focused testing procedures, which showed isometric exercises to be ineffective in reducing the incidence of concussion [30, 33, 48, 53, 55]. The “golden thread” of these findings may be a move away from testing muscles in isolation and towards testing closed kinematic chains (multi-segment force transmission) to dissipate forces to the brain [63–65].

The findings of this research come at a time of increased global attention on concussion prevention to improve welfare of participants involved in sport.

## Limitations

This scoping review included articles published in English language only, due to resource and budget constraints. In doing so, it is possible that articles in other languages that may have met the study’s inclusion criteria were excluded in the search strategy. A second limitation is that a timeframe of the past 15 years was used, which may have excluded older articles that met the inclusion criteria. Although the available evidence is limited, the use of the scoping review methodology, rather than a systematic review, allowed for discussion and analysis of a broad spectrum of relevant studies with varied methodologies.

## Conclusion

The results of this scoping review are presented at a time of an increased global scrutiny of concussion prevention to improve the welfare of participants involved in sport and reveal a dearth of literature addressing physical preparation strategies to reduce or prevent the incidence of concussion. The small amount of research in this area has shown conflicting results for modifiable risk factors relating to neck size, neck strength, and neck stiffness. Future research should assess the effects of both proprioceptive and dynamic strengthening of the neck and surrounding musculature to reduce forces transmitted to the brain or to increase resilience during participation in sports. The potential of effective protective mechanisms to reduce the incidence and the effects of concussion warrants further research.

## Supplementary Information

**Additional file 1.** Appendix A.

**Additional file 2.** Appendix B.

## Data Availability

All data generated or analyzed during this study are included in this published article.

## Abbreviations

SRC: Sport-related concussion
IMVC: Isometric maximum voluntary contraction
IG: Intervention group
CG: Control group
DUHNM: Duke University Head Neck Model
NCAC: National Crash Analysis Center
EMG: Electromyographic
SCM: Sternocleidomastoid
SSC: Semispinalis capitis
UT: Upper trapezius
RM: Repetition maximum
