# Supplementary material for: Physical Conditioning Strategies for the Prevention of Concussion in Sport: a Scoping Review
Source: Sports Med Open. 2021 May 17;7:31. doi: 10.1186/s40798-021-00312-y (PMC8128965; doi:10.1186/s40798-021-00312-y)
Supplement: Supplementary file 1 — Additional file 1. Appendix A. [file 40798_2021_312_MOESM1_ESM.docx]

Appendix A

The search strategy of UKZN’s electronic databases (PubMed; WorldCat.org and Mendeley) used the keywords and combinations of keywords as described below:

Your Search: ((((("sports"[MeSH Terms] OR "sports"[All Fields]) AND ("prevention and control"[Subheading] OR ("prevention"[All Fields] AND "control"[All Fields]) OR "prevention and control"[All Fields] OR "prevention"[All Fields])) AND ("brain concussion"[MeSH Terms] OR ("brain"[All Fields] AND "concussion"[All Fields]) OR "brain concussion"[All Fields] OR "concussion"[All Fields])) AND ("prevention and control"[Subheading] OR ("prevention"[All Fields] AND "control"[All Fields]) OR "prevention and control"[All Fields] OR "prevention"[All Fields])) AND ("sports"[MeSH Terms] OR "sports"[All Fields])) AND ("brain concussion"[MeSH Terms] OR ("brain"[All Fields] AND "concussion"[All Fields]) OR "brain concussion"[All Fields]) Sort: Library. Group related editions: on. Year: 2009 – 2019. Databases: UKZN Libraries; PubMed; WorldCat.org; WorldCat; Mendeley. Content: Peer Reviewed.
