# Supplementary material for: Physical Conditioning Strategies for the Prevention of Concussion in Sport: a Scoping Review
Source: Sports Med Open. 2021 May 17;7:31. doi: 10.1186/s40798-021-00312-y (PMC8128965; doi:10.1186/s40798-021-00312-y)
Supplement: Supplementary file 2 — Additional file 2. Appendix B. [file 40798_2021_312_MOESM2_ESM.docx]

Appendix B

The search strategies for University of Pretoria’s electronic databases.

| Database | Search string and keywords |
| --- | --- |
| WorldCat | brain concussion OR  concussions AND  sports AND  (prevention or intervention or treatment or program ) |
| EBSCOhost Research Databases Advanced Search; Africa-Wide; Academic Search CINAHL;MEDLINE;SPORTDiscus with Full Text | brain concussion OR concussions AND sports AND ( prevention or intervention or treatment or program ) |
| Ovid MEDLINE(R) without Revisions | 1     exp *Brain Concussion/pc [Prevention & Control] 2     exp *Sports/  3     1 and 2  4     limit 3 to (english language and yr="2005 -Current") |
